# The impact of maintenance therapy on sleep-wake rhythms and cancer-related fatigue in pediatric acute lymphoblastic leukemia

**DOI:** 10.1007/s00520-020-05444-7

**Published:** 2020-04-13

**Authors:** L. M. H. Steur, G. J. L. Kaspers, E. J. W. van Someren, N. K. A. van Eijkelenburg, I. M. van der Sluis, N. Dors, C. van den Bos, W. J. E. Tissing, M. A. Grootenhuis, R. R. L. van Litsenburg

**Affiliations:** 1 Emma Children’s Hospital, Amsterdam UMC, Pediatric Oncology, Cancer Center Amsterdam, Vrije Universiteit Amsterdam, ZH 8D12, P.O. Box 7057, 1007 MB Amsterdam, The Netherlands; 2 Princess Máxima Center for Pediatric Oncology, Heidelberglaan 25, 3584 CS Utrecht, The Netherlands; 3 Dutch Childhood Oncology Group, Utrecht, The Netherlands; 4 Department of Sleep and Cognition, Netherlands Institute for Neuroscience (an institute of the Royal Netherlands Academy of Arts and Sciences), Amsterdam, The Netherlands; 5 Department of Integrative Neurophysiology, Amsterdam Neuroscience, Center for Neurogenomics and Cognitive Research (CNCR), VU University Amsterdam, Amsterdam, The Netherlands; 6 Psychiatry, Amsterdam Neuroscience, Amsterdam UMC, Vrije Universiteit Amsterdam, Amsterdam, The Netherlands; 7 Department of Pediatric Oncology, Sophia Children’s Hospital, Erasmus Medical Center, Rotterdam, the Netherlands; 8 Department of Pediatric Oncology, Amalia Children’s Hospital, Radboud University Medical Center, Nijmegen, the Netherlands; 9 Department of Pediatric Oncology, Emma Children’s Hospital, Amsterdam UMC, Academic Medical Center, Amsterdam, the Netherlands; 10 Department of pediatric oncology, University Medical Center Groningen, University of Groningen, Groningen, The Netherlands

**Keywords:** Sleep, Cancer-related fatigue, Acute lymphoblastic leukemia, Pediatric, Actigraphy, Dexamethasone

## Abstract

**Purpose:**

To assess the impact of maintenance therapy and the additional impact of dexamethasone treatment on cancer-related fatigue and sleep-wake rhythms in pediatric acute lymphoblastic leukemia (ALL) patients and to determine the association between these outcomes.

**Methods:**

A national cohort of pediatric ALL patients (≥ 2 years) was included (± 1 year post-diagnosis). Patients receiving dexamethasone were assessed twice (assessment with and without dexamethasone). Actigraphy assessments were used to calculate sleep-wake outcomes with nonparametric methods. Cancer-related fatigue was assessed with the PedsQL Multidimensional Fatigue Scale. Sleep-wake rhythms and cancer-related fatigue were compared between patients participating in the assessment without dexamethasone and healthy children (linear regression) and between assessments with and without dexamethasone (mixed models). Using linear regression, associations between sleep-wake outcomes and cancer-related fatigue were determined during assessments with and without dexamethasone.

**Results:**

Responses were collected for 125 patients (113 assessments with and 81 without dexamethasone). The sleep-wake rhythm was less stable (*p* = 0.03) and less robust (*p* = 0.01), with lower physical activity levels (*p* < 0.001) and higher cancer-related fatigue levels (*p* < 0.001) in ALL patients compared to healthy children. Physical activity was lower (*p* = 0.001) and cancer-related fatigue more severe (*p* ≤ 0.001) during assessments with dexamethasone compared to without dexamethasone. Sleep-wake outcomes were significantly associated with cancer-related fatigue during periods without dexamethasone, but not during periods with dexamethasone.

**Conclusion:**

Sleep-wake rhythms are disturbed, physical activity levels lower, and cancer-related fatigue levels higher during maintenance therapy. Interventions aimed to enhance sleep-wake rhythms during maintenance therapy could improve cancer-related fatigue. Families should be supported in coping with the additional burden of dexamethasone treatment to improve well-being of ALL patients.

**Electronic supplementary material:**

The online version of this article (10.1007/s00520-020-05444-7) contains supplementary material, which is available to authorized users.

## Introduction

Acute lymphoblastic leukemia (ALL) is the most common type of pediatric cancer, and treatment is long and intensive. After a first, intensive induction phase aimed to achieve complete remission, the vast majority of patients receive maintenance therapy until 2 to 3 years after initial diagnosis to prevent relapse. Maintenance is a relative stable phase in which most patients resume their normal daily activities. However, maintenance therapy still consists of frequent intravenous and/or oral chemotherapy administrations and treatment-related toxicities and hospital admissions are common [1]. Moreover, maintenance therapy includes cyclic glucocorticoids treatment in the majority of patients. Glucocorticoids, and particularly dexamethasone, are known for their neurobehavioral side effects, among which are sleep disturbances [2–5].

Pediatric patients with ALL during maintenance are vulnerable to disturbed sleep and sleep-wake rhythms for several reasons. Several physical (treatment-related toxicities such as pain or fever/infectious diseases), psychosocial (for example anxiety) and social stressors (for example impaired school attendance and changed family routines) during maintenance treatment might hamper sleep quality and quantity, as well as sleep hygiene. Changed parenting strategies, such as more lax parenting, pampering, and bribing, have been described in childhood oncology populations and could impede sleep hygiene [6–8]. The additional effect of dexamethasone treatment during maintenance treatment may also cause sleep and sleep-wake rhythm disturbances. The underlying mechanisms of sleep problems and sleep-wake rhythm disturbances during dexamethasone treatment are not yet fully understood. The unbalanced glucocorticoid receptor (GR) and mineralocorticoid receptor (MR) occupation in the brain has been described previously as a potential cause of dexamethasone-induced neurobehavioral side effects [2, 9, 10]. The hypothalamus-pituitary-adrenal axis (HPA axis) is important for sleep through the synthesis of cortisol [11, 12]. Previous studies described an association between cortisol and sleep and sleep-wake rhythm outcomes [12–14]. In the brain, cortisol binds both the MR and the GR [11]. Cortisol binding to the MR and GR stimulates slow wave sleep and inhibits rapid eye movement sleep [11]. Compared to cortisol, dexamethasone has a higher affinity for the GR, whereas it does not bind the MR in the brain [2, 10]. Through a negative feedback loop acting on the HPA axis, dexamethasone suppresses endogenous cortisol production. This leads to (over)activation of the GR and complete endogenous ligand depletion or lower occupancy of the MR in the brain [10]. Kellner et al. reported that endogenous receptor occupancy and regulation seem to be important for the effect of the MR on sleep [15]. Therefore, dexamethasone-induced depletion of cortisol and the subsequent unbalanced receptor binding in the brain may play a role in the etiology of sleep and sleep-wake rhythm disturbances in patients treated with dexamethasone. Additionally, the (behavioral) side effects of dexamethasone, such as excessive eating, aggressive moods, and depressive symptoms, may directly interfere with appropriate sleep hygiene and thereby impair sleep and sleep-wake rhythmicity [3, 5].

While sleep disturbances such as nighttime awakenings, sleep anxiety, bedtime resistance, and parasomnias have previously been described, evidence on sleep-wake rhythms during pediatric ALL maintenance therapy is scarce [16–18]. Only a single study in pediatric ALL patients described impaired sleep-wake rhythmicity after dexamethasone initiation [19]. However, since sleep-wake rhythms were not compared to healthy children in this previous study, it is not yet known whether sleep-wake rhythms are actually disturbed.

Cancer-related fatigue is one of the most common side effects of anticancer treatments, and increased severity has been described during dexamethasone treatment [20–22]. Cancer-related fatigue is a disabling symptom that often continues after cessation of treatment [23]. It impairs school functioning and reduces the ability to participate in social roles and activities [24, 25]. It is a multidimensional problem, but the etiology is not yet fully understood [20]. A biopsychosocial model including demographic, biological, medical, functional, and behavioral factors contributing to cancer-related fatigue seems likely [20]. A relationship between sleep-wake rhythm disturbances and cancer-related fatigue has previously been described in pediatric oncology populations [19, 26]. This suggests that the sleep-wake rhythm plays a role in the etiology of cancer-related fatigue.

Given the high burden of maintenance treatment and its potential implications for the sleep-wake rhythm and cancer-related fatigue, it is important to better understand these relationships in pediatric ALL patients. This might provide opportunities for interventions to increase well-being of ALL patients.

Therefore, this study aimed to (1) compare sleep-wake rhythms and cancer-related fatigue of pediatric ALL patients during maintenance therapy to healthy children; (2) determine the additional effect of dexamethasone treatment on these outcomes; and (3) assess the association between sleep-wake outcomes and cancer-related fatigue.

## Methods

The results described here are part of the SLAAP [*SLEEP*] study (SLeep in children with Acute lymphoblastic leukemia And their Parents), an observational, longitudinal, multicenter study on sleep, sleep-wake rhythms, quality of life and cancer-related fatigue in pediatric ALL patients and functioning of their parents. Results on sleep-wake rhythms and cancer-related fatigue measured at 1 year post-diagnosis are reported here.

Patients were identified through the Dutch Childhood Oncology Group (DCOG) registry that includes all pediatric patients with a diagnosis of cancer in the Netherlands. Patients were eligible if they were (1) diagnosed with primary ALL and treated according to the national first-line DCOG treatment protocol ALL-11, open to patients aged 1 to 19 years, and (2) ≥ 2 years of age at assessment. Furthermore, parents and patients needed to master Dutch sufficiently to complete the questionnaires. Patients were recruited in the following Dutch pediatric oncology centers: Emma Children’s Hospital/Academic Medical Center and VU University Medical Center Amsterdam, Wilhelmina’s Children’s Hospital/University Medical Center Utrecht, Princess Máxima Center for pediatric oncology Utrecht, Sophia Children’s Hospital/Erasmus Medical Center Rotterdam, Beatrix Children’s Hospital/University Medical Center Groningen, Amalia Children’s Hospital/Radboud University Medical Center Nijmegen. Parents and patients (≥ 12 years) provided informed consent for participation. The Institutional Review Board of the Erasmus Medical Center approved this study.

The study assessments were planned around 1 year after diagnosis, during maintenance therapy. During this phase, ALL treatment intensity depends on risk group stratification. Patients were stratified to the following risk groups, with increasing treatment intensity, based on response to treatment and cytogenetics: standard risk (SR), medium risk (MR) and high risk (HR). SR maintenance therapy consisted of daily oral mercaptupurine and weekly oral methotrexate. During the study assessment, maintenance therapy for MR group patients consisted of continuous oral mercaptupurine, weekly intravenous methotrexate administrations, 3-weekly vincristine administrations, and cyclic oral dexamethasone treatment (21-day cycles: 5 days with dexamethasone alternated with 16 days without dexamethasone). In addition, MR patients received intrathecal chemotherapy around once per 4 months. Assessments were planned during a week without intrathecal chemotherapy. Only one HR patient completed the study and was excluded from the analyses described here because of the higher treatment intensity for HR patients compared to SR and MR patients.

A single assessment was planned for SR patients. Two assessments were planned for MR patients: (1) during a period with dexamethasone and (2) during a period without dexamethasone. Participants were instructed to start the actigraphy assessment during the period with dexamethasone the first day of the 5-day dexamethasone treatment and the assessment without dexamethasone 7 day before start of a following dexamethasone treatment cycle. Participants were instructed to complete the questionnaires (regarding functioning during the assessment week) at a self-chosen day during that week. To reduce the burden to the families, assessments were preferably planned with at least one 21-day treatment cycle in between. Whether the first assessment was during a period with or without dexamethasone was based on patient/parent preference. The assessments, including questionnaires and actigraphic recordings, took place at home.

### Measures

#### Sociodemographic and medical information

Parents filled out a survey to collect the following sociodemographic information: patient and parental age and sex, child’s sleep medication use (yes or no), and parental highest attained educational level. Educational level was defined according to Statistics Netherlands [27] and dichotomized as lower (low and middle educational level) versus higher (high educational level). Information on time since diagnosis and risk group stratification was collected through the DCOG.

#### Actigraphy-derived sleep-wake rhythm

Actigraphy assessments were used to calculate sleep-wake rhythm outcomes. An actigraph (ActiGraph wGT3X-BT, Pensacola, FL, USA) is a nonintrusive device that measures the occurrence and intensity of limb movements. Patients were instructed to wear the actigraph on their wrist for 24 h for 7 days. Actigraphy has been validated against polysomnography and has proven to be an adequate method to measure sleep-wake patterns in infants, children, and adolescents [28–30]. Participants were instructed to record sleep-wake schedule information in a sleep log to facilitate correct interpretation of the actigraphy data. Invalid data, defined as probable non-wear time ≥ 3 consecutive hours, was identified [31]. A 24-h period starting at the onset of this non-wear time was then removed from further analysis [31]. Sleep-wake outcomes were calculated if valid data was available for at least 72 h [32].

There are different approaches to quantify sleep-wake actigraphic recordings. Two commonly used methods are the cosinor analysis and the nonparametric method. Cosinor analysis is parametric and presumes that the activity level variation over the day is best described with a 12:12 h symmetrical sinusoidal. However, the sleep-wake rhythm, particularly in children, is far from symmetrical and sinusoidal. To better accommodate the non-sinusoidal, asymmetric activity pattern of everyday life, nonparametric methods have been proposed, that do not make assumptions about the distribution of the rhythm. Accordingly, the nonparametric method seems to describe the sleep-wake pattern more accurately than cosinor analysis [32, 33]. Nonparametric methods were used to obtain the following sleep-wake rhythm variables (definitions are provided in Table 1): interdaily stability (IS), intradaily variability (IV), M5 counts, M10 counts, and relative amplitude (RA) [33].

**Table 1:** Definitions of the sleep-wake rhythm variables

Variable	Range	Definition
Interdaily stability (IS)	0–1	An estimate of the stability of the rhythm, and describes the synchronization of the rhythm, wherein 1 signifies a perfect synchronization to the dark-light cycle
Intradaily variability (IV)	0–2	An estimate of the 24-h rest-activity rhythm and reflects the fragmentation of the rhythm, a higher IV indicates a more fragmented rhythm
L5 counts	0–∞	Activity counts (physical activity) during the least active 5 h of the day.
M10 counts	0–∞	Activity counts (physical activity) during the most active 10 h of the day.
Relative amplitude (RA)	0–1	Ratio of the difference and the sum of M10 and L5 counts. A higher RA indicates a bigger difference between the least and most active period during the day, hence a better sleep-wake rhythm.

For comparison, sleep-wake rhythm variables were also obtained in healthy children, aged 2–18 years with the same type of actigraph [34]. Recruitment took place through snowball sampling and word of mouth referrals within the professional and social network of the research team. Children were not eligible if they visited a health care provider for sleep disturbances in the preceding 3 months, used any type of sleep medication (including melatonin), or had a medical condition that could potentially affect sleep or the circadian rhythm (epilepsy, blindness, exacerbation of asthma or severe eczema). Additionally, children were excluded if they or their parents were insufficiently fluent in Dutch. Actigraphy data was collected in the same manner as in patients with ALL. Valid actigraphy data was available for 85 healthy children (median age 8.5 years [interquartile range 5.5–15.3], 50.6% boys, highest parental educational level: 34.1% lower educational level and 65.9% higher educational level).

#### Cancer-related fatigue

The Dutch parent-proxy version of the PedsQL Multidimensional Fatigue Scale (PedsQL MFS) was used to assess cancer-related fatigue [35, 36]. This 18-item questionnaire allows for an overall fatigue score and three subscale scores: general fatigue, sleep-rest fatigue and cognitive fatigue. The occurrence of problems is assessed over the past week on a 5-point Likert scale. Items are rescored to a 0–100 scale. A higher score indicates less cancer-related fatigue. The subscale scores were used in this study. Scores in healthy Dutch children have previously been collected by Gordijn et al. [35]. The internal consistency of the subscales was adequate (Cronbach’s alpha > 0.70) in the population of healthy children as well as in the study population. The questionnaire was completed paper-pencil or through a secured online web portal depending on parent preference.

### Statistical analysis

Data from the assessments in MR patients during the period without dexamethasone and in SR patients were analyzed as one group to represent the ALL population during maintenance therapy that participated in the assessment without dexamethasone. Additional analyses showed that there were no statistically significant differences in sleep-wake rhythms and cancer-related fatigue between these patients (Supplemental Table S1). Data from the assessment with dexamethasone in MR patients were analyzed to reflect the additional burden of dexamethasone treatment in MR patients.

Descriptive statistics of socio-demographics and medical factors are presented for patients that participated in the assessment without dexamethasone (including SR and MR patients during periods without dexamethasone) and MR patients that participated in the assessment with dexamethasone separately.

Sleep-wake outcomes and cancer-related fatigue scores were presented for both patient groups and healthy children. Outcomes of assessments without dexamethasone were compared to healthy children using linear regression models. Regression models were adjusted for patient age, sex, sleep medication use, and parental educational level as differences in these outcomes between patients with ALL and healthy children could affect sleep-wake rhythm outcomes and cancer-related fatigue. In addition, the regression models for cancer-related fatigue were adjusted for parental sex, since parent-reports may differ between fathers and mothers.

Additionally, sleep-wake outcomes and cancer-related fatigue scores were compared between patients that participated in the assessment without dexamethasone and MR patients that participated in the assessment with dexamethasone. Mixed model analyses were used to correct for the dependency of MR patients that participated in both the assessment with and without dexamethasone. Analyses were adjusted for patient age, sex, sleep medication use, time since diagnosis, and parental educational level.

Associations between sleep-wake outcomes and cancer-related fatigue were evaluated with linear regression models for assessments with and without dexamethasone separately. Regressions models were adjusted for patient age, sex, sleep medication use, risk group stratification, time since diagnosis, and parental educational level.

## Results

**Fig. 1:**
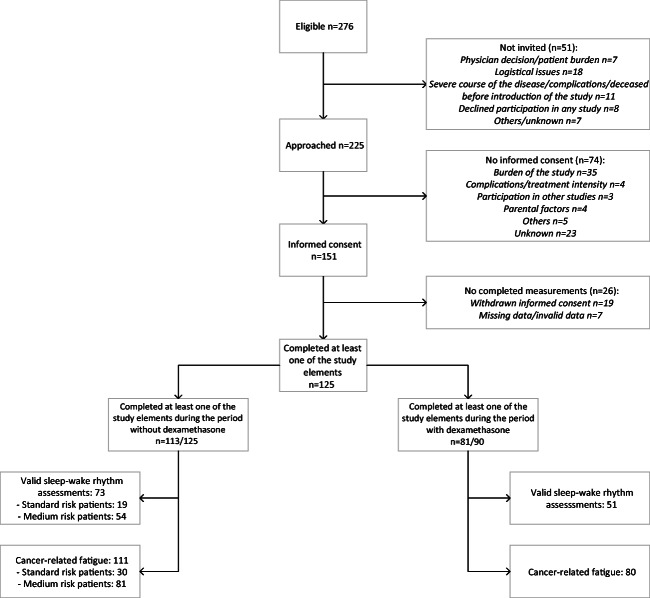
Patient enrollment

Details regarding the patient enrollment are described in Fig. 1. Informed consent was provided for 151 patients, of whom 125 completed at least one of the study assessments described here. The assessment without dexamethasone was completed by 113 patients, including 30 SR and 83 MR patients. They completed 73 valid sleep-wake rhythm assessments and 111 parent-reports on cancer-related fatigue. The assessment with dexamethasone was completed by 81 out of 90 eligible MR patients, who completed 51 valid sleep-wake rhythm assessments and 80 cancer-related fatigue parent-reports.

Patient and parental socio-demographics and patient medical factors were comparable for patients that participated in the assessments without dexamethasone and for MR patients that participated in the assessment with dexamethasone (Table 2).

**Table 2:** Baseline characteristics for patients with ALL during periods with and without dexamethasone

	**Patients participating in the assessment without dexamethasone**^a^ (***n* = 113**)	**Patients participating in the assessment with dexamethasone** (***n* = 81**)
*Patient- and treatment-related factors*		
Patient age at diagnosis in years, median [IQR]	4.8 [3.0–8.7]	5.0 [3.0–9.6]
Female patient sex, *N* (%)	50 (44.2)	33 (40.6)
Risk group stratification, *N* (%)		
Standard risk group	30	–
Medium risk group	83	81 (100)
Time since diagnosis in months, median [IQR]	13.5 [12.7–14.0]	12.8 [12.4–13.5]
Use of sleep medication, *N* (%)	14 (12.4)	9 (11.1)
*Parental- and family factors*		
Parental age in years, mean ± SD	38.9 (6.1)	39.5 (6.9)
Female parental sex, *N* (%)	91 (80.5)	63 (77.8)
Educational level, *N* (%)^b^		
Low	3 (2.7)	2 (2.5)
Middle	29.0 (25.7)	20 (24.7)
High	77 (68.1)	55 (67.9)
Unknown	4 (3.5)	4 (4.9)

*N* number, *SD* standard deviation, *IQR* interquartile range, *ALL* acute lymphoblastic leukemia

^a^Include both standard and medium risk group patients

^b^Low educational level = no education, primary school, lower secondary education; middle educational level = upper secondary education, pre-university education, intermediate vocational education; high educational level = higher vocational education, university

### Sleep-wake outcomes

Descriptive statistics of sleep-wake outcomes of ALL patients and healthy children are shown in Table 3. IS (*B* = − 0.04, *p* = 0.030), M10 counts (*B* = − 412.23, *p* < 0.001), and RA (*B* = − 0.02, *p* = 0.012) were significantly lower in ALL patients participating in the assessment without dexamethasone compared to healthy children, reflecting a less stable and less robust rhythm, and lower physical activity in ALL patients (Supplemental Table S2). IV and L5 counts were not significantly different from healthy children. During assessments without dexamethasone, M10 counts were significantly higher (*B* = 254.94, *p* = 0.001) compared to assessment with dexamethasone in MR patients (Supplemental Table S3). Other sleep-wake outcomes were not different between assessments with and without dexamethasone.

**Table 3:** Sleep-wake rhythm variables and cancer-related fatigue in patients with ALL and in healthy children

	**Patients participating in the assessment without dexamethasone**^a^ ***(n* = 73)**	**Patients participating in the assessment with dexamethasone (*n* = 51)**	**Healthy children** **(n = 85)**
*Sleep-wake rhythm variables*	*Mean ± SD and median [IQR]*	*Mean ± SD and median [IQR]*	*Mean ± SD and median [IQR]*
Interdaily stability	0.61 ± 0.11	0.59 ± 0.12	0.62 ± 0.13
	0.64 [0.53–0.69]	0.60 [0.51–0.68]	0.63 [0.55–0.71]
Intradaily variability	0.74 ± 0.21	0.75 ± 0.21	0.72 ± 0.20
	0.70 [0.57–0.85]	0.70 [0.61–0.88]	0.69 [0.58–0.83]
L5 counts	53.22 ± 25.13	57.56 ± 25.81	51.85 ± 23.95
	48.92 [34.88–64.53]	53.66 [37.67–68.63]	46.32 [36.25–63.50]
M10 counts	1892.79 ± 662.09	1524.26 ± 600.23	2187.10 ± 709.33
	1992.12 [1573.08–2349.36]	1428.77 [1112.88–1969.25]	2244.55 [1520.47–2658.15]
Relative amplitude	0.93 ± 0.07	0.92 ± 0.05	0.95 ± 0.03
	0.94 [0.93–0.97]	0.93 [0.89–0.96]	0.96 [0.94–0.97]
	**Patients participating in the assessment without dexamethasone**^a^ ***(n* = 111)**	**Patients participating in the assessment with dexamethasone** **(n = 80)**	**Healthy children** ***(n = 497)***^34^
*Cancer-related fatigue*	*Mean ± SD and median [IQR]*	*Mean ± SD and median [IQR]*	*Mean ± SD and median [IQR]*
General fatigue	62.76 ± 22.11	50.13 ± 23.19	81.27 ± 14.17
	62.50 [50.00–75.00]	45.83 [33.33–66.67]	83.33 [70.83–91.67]
Sleep-rest fatigue	71.51 ± 19.79	60.49 ± 17.94	83.84 ± 13.86
	75.00 [58.33–87.50]	59.17 [46.88–73.96]	87.50 [75.00–95.83]
Cognitive fatigue	78.75 ± 18.99	71.04 ± 20.70	78.48 ± 17.93
	75.00 [66.67–95.83]	75.00 [58.33–91.67]	79.17 [66.67–95.83]

*ALL* acute lymphoblastic leukemia, *SD* standard deviation, *IQR* interquartile range, *N* number

^a^Include both standard and medium risk group patients

### Cancer-related fatigue

Descriptive statistics of cancer-related fatigue scores of patients and healthy children are shown in Table 3. General (*B* = − 17.52, *p* < 0.001) and sleep-rest (*B* = − 12.11, *p* < 0.001) fatigue scores were significantly lower (reflecting higher levels of cancer-related fatigue) in patients participating in the assessment without dexamethasone compared to healthy children (Supplemental Table S2). Cancer-related fatigue scores were significantly higher, indicating lower levels of cancer-related fatigue, during assessments without dexamethasone compared to assessments with dexamethasone (general (*B* = 10.50, *p* = 0.001), sleep-rest (*B* = 10.63, *p* < 0.001), and cognitive fatigue (*B* = 9.63, *p* = 0.001)) (Supplemental Table S3).

### Association between sleep-wake outcomes and cancer-related fatigue

During assessments without dexamethasone, higher M10 counts (higher physical activity) and higher RA (more robust rhythm) were significantly associated with lower levels of cancer-related fatigue (all subscales). Lower IV, indicating a less fragmented rhythm, was significantly associated with less sleep-rest fatigue (Table 4). Higher L5 counts, reflecting higher activity during the 5 least active hours of the day, were associated with more sleep-rest fatigue (Table 4). Associations were not statistically significant for MR patients during periods with dexamethasone.

**Table 4:** Association between sleep-wake rhythm variables (actigraphy derived) and cancer-related fatigue (PedsQL MFS scores) in patients with ALL

**General fatigue *B*** **(95% CI)**^a^		**Sleep-rest fatigue** ***B*** **(95% CI) **^a^	**Cognitive fatigue** ***B*** **(95% CI) **^a^
Patients participating in the assessment without dexamethasone (*n* = 69)			
Interdaily stability (0.1 change)	4.47 (− 1.05; − 10.00)	6.85 (2.12; 11.57)**	1.30 (−3.60; 6.20)
Intradaily variability (0.1 change)	− 4.87 (− 7.81; 1.93)**	− 4.63 (− 7.22; − 2.03)**	− 3.05 (− 5.72; − 3.81)**
L5 counts (10 counts change)	− 1.83 (− 4.06; 0.41)	− 2.11 (− 4.08; − 0.14)**	− 0.54 (− 2.53; 1.44)
M10 counts (10 counts change)	0.22 (0.14; 0.31)*	0.22 (0.15; 0.29)*	0.14 (0.06; 0.22)**
Relative amplitude (0.1 change)	14.55 (6.96; 22.14)*	17.12 (10.97; 23.27)*	9.06 (2.08; 16.05)**
Patients participating in the assessment with dexamethasone (*n* = 47)			
Interdaily stability (0.1 change)	3.38 (− 3.80; 10.56)	4.61 (− 0.89; 10.12)	− 4.29 (− 11.30; 2.72)
Intradaily variability (0.1 change)	2.56 (− 1.26; 6.37)	− 0.91 (− 3.97; 21.39)	1.34 (− 2.29; 51.56)
L5 counts (10 counts change)	− 0.89 (− 3.57; 1.79)	− 1.34 (− 3.41; 0.74)	− 0.64 (− 3.23; 1.94)
M10 counts (10 counts change)	0.04 (− 0.11; 0.18)	0.05 (− 0.06; 0.17)	− 0.01 (− 0.02; 0.01)
Relative amplitude (0.1 change)	8.60 (− 7.98; 25.18)	10.34 (− 2.45; 23.12)	− 1.10 (− 17.26; 15.07)

*ALL* acute lymphoblastic leukemia, *CI* confidence interval, *MFS* Multidimensional Fatigue Scale, *N* number, *L5 counts* activity counts during the least active 5 h of the day, *M10 counts* activity counts during the most active 10 h of the day

Significant associations are italicized; *p value < 0.05; **p value < 0.001

^a^Adjusted for child’s age, sex and current sleep medication use, risk group stratification, time since diagnosis and highest attained parental educational level

^b^Adjusted for child’s age, sex and current sleep medication use, time since diagnosis and highest attained parental educational level

## Discussion

During maintenance therapy, pediatric patients with ALL demonstrated disturbed sleep-wake rhythms, lower physical activity levels, and increased levels of cancer-related fatigue compared to healthy children. During dexamethasone periods, patients showed even lower physical activity and more severe cancer-related fatigue.

The reduced stability and robustness of the sleep-wake rhythm in ALL patients during maintenance therapy may have several reasons. First, to align the circadian sleep-wake cycle to the environmental 24-h light-dark cycle it needs to be synchronized by external cues, such as light, scheduled sleep, physical activity, and meals [37–40]. Several factors during ALL maintenance therapy can alter these external cues and thereby hamper appropriate synchronization of the sleep-wake rhythm. Treatment-related toxicities, psychological stressors, and environmental noises during hospitalization may, for example, influence light exposure, physical activity, social activities, and school attendance. Second, more lenient parenting that has been described in ALL patients may impair limit setting regarding sleep hygiene, subsequently resulting in sleep-wake rhythm disturbances [6, 7].

Daytime napping and night awakenings have been described in pediatric patients with ALL, which may reflect in fragmentation of the sleep-wake rhythm. Considering the physical and psychosocial stressors related to ALL treatment, increased fragmentation of the rhythm was expected [16, 22, 41]. However, fragmentation of the sleep-wake rhythm was not increased in the current study. This is probably the result of the low overall physical activity levels in our sample. Moreover, consistent with our results, intradaily variability was within normal limits in hospitalized children with central nervous system cancer [26].

More disturbed sleep-wake rhythms were expected during dexamethasone treatment as has been described previously [19]. However, except for lower physical activity levels, sleep-wake rhythms were not different between assessments with and without dexamethasone. We hypothesized that the unbalanced glucocorticoid receptor occupancy plays a role in the etiology of sleep-wake rhythm disturbances during dexamethasone treatment [2, 9–11]. However, based on the short half-life time (1.27 h) of oral dexamethasone, the receptor occupancy would expected to be restored during the intervals without dexamethasone [42]. The biological effect of dexamethasone might still precipitate sleep-wake rhythm disturbances. Perpetuating factors may subsequently cause longer term sleep-wake rhythm disturbances. For example, the emotional and behavioral side-effects of dexamethasone may induce structural behavioral changes. These behavioral changes might in turn result in adapted parenting strategies that have previously been described and changed family routines [6, 7]. The behavioral changes and changed family structures do probably not resolve in the short intervals between consecutive dexamethasone cycles and hamper reversibility of sleep-wake rhythm disturbances in-between dexamethasone cycles.

Higher levels of cancer-related fatigue were reported for patients participating in assessments without dexamethasone compared to healthy children, with the exception of cognitive fatigue. The absence of cognitive fatigue is in line with a previous study in adolescent cancer patients and is probably the result of lower cognitive demands and expectations during ALL treatment [43]. Furthermore, we confirmed the previously described increased cancer-related fatigue levels during periods with dexamethasone [22]. In patients participating in the assessments without dexamethasone, sleep-wake rhythm disturbances were significantly associated with more severe cancer-related fatigue. The absence of this relationship during assessments with dexamethasone indicates that the additional cancer-related fatigue during periods with dexamethasone is independent of the sleep-wake rhythm. The additional cancer-related fatigue during assessments with dexamethasone may have several reasons. First, the group of patients that participated in the assessment without dexamethasone also includes SR patients, whereas patients participating in the assessment with dexamethasone were MR patients only. The higher levels of cancer-related fatigue during periods with dexamethasone may reflect the higher treatment burden for MR patients (weekly hospital visits, intravenous methotrexate, and 3-weekly vincristine administration). However, no differences were found between MR patients during periods without dexamethasone and SR patients, but the small number of SR patients could have limited the power to find a significant difference. Second, the depressive symptoms and the associated lethargy described during dexamethasone may be perceived as cancer-related fatigue [5]. Third, parental demands are higher during dexamethasone given the many neurobehavioral side-effects which may result in a higher parent perceived burden and overreporting of symptoms.

Given the impact of maintenance treatment on sleep-wake rhythms and cancer-related fatigue, clinicians should pay attention to these outcomes and families should be supported in coping with this in general, and with the additional burden of dexamethasone treatment in particular. Implementation of interventions to restore healthy sleep-wake rhythms could provide opportunities to improve cancer-related fatigue during maintenance therapy. In a previous study in pediatric patients with ALL, hydrocortisone addition to dexamethasone treatment, to achieve a more balanced receptor occupancy, reduced neurobehavioral side effects in patients with severe behavioral and sleep problems [2]. A validation study in patients with severe behavioral and sleep problems is currently ongoing. Based on the hypothesis that sleep-wake rhythm disturbances during maintenance treatment are precipitated by the unbalanced receptor occupancy, hydrocortisone might also be an effective intervention to prevent sleep-wake rhythm disturbances. Furthermore, interventions to encourage physical activity could enhance sleep-wake rhythms and have proven to be feasible in pediatric patients with ALL [44]. Finally, the reduced stability and robustness of the sleep-wake rhythm might result from impaired sleep hygiene during maintenance therapy. Systematic monitoring of sleep and attention to sleep hygiene during ALL treatment may provide opportunities for early interventions to prevent sleep-wake rhythm disturbances. Knowledge on healthy child sleep in parents is generally poor. Educational interventions have proven to effectively improve knowledge in parents of healthy children [45, 46]. Such educational interventions could potentially improve sleep hygiene in pediatric patients with ALL and thereby enhance sleep-wake rhythms. Psycho-education should include information on healthy sleep habits and address incorrect cognitions on sleep. Information on physical activity should be incorporated to encourage low intensity physical activity, within the limits of the physical condition of the child. Moreover, information on neurobehavioral side effects of dexamethasone could support parents in coping with these side effects. Additionally, providing eye mask and ear plugs during hospitalization could reduce the environmental impact on sleep-wake rhythms [47].

Some limitations of this study need to be mentioned. First, not all patients participated in all study assessments. Hence, selection bias and participation bias, for example based on treatment-related toxicity, cannot completely be ruled out. The study may therefore have underestimated sleep-wake rhythm disturbances and cancer-related fatigue. Second, participants were instructed to start the actigraphy assessment during the period with dexamethasone on the first day of dexamethasone treatment. However, it was not registered which day of the dexamethasone cycle participants actually started the assessment. Since sleep-wake rhythm assessments lasted 7 days, we could have measured washout effects of dexamethasone. Third, self-reported cancer-related fatigue was not taken into account while disagreement between parent- and self-reports has been reported in childhood oncology [48]. Finally, since a lower socio-economic status has been associated with less healthy sleep behaviors, the overrepresentation of highly educated families in our study may have underestimated the prevalence and severity of disturbed sleep-wake rhythms [27, 49].

In conclusion, sleep-wake rhythms were disturbed and cancer-related fatigue levels were increased in pediatric patients with ALL during maintenance therapy. During periods with dexamethasone patients showed even lower physical activity and higher levels of cancer-related fatigue. More disturbed sleep-wake rhythms were associated with higher levels of cancer-related fatigue during periods without dexamethasone. Interventions aimed to enhance sleep-wake rhythms and support of families in coping with sleep-wake rhythm disturbances and with the additional burden of dexamethasone treatment in particular could improve well-being of ALL patients and their families during maintenance therapy.

## Electronic supplementary material

ESM 1 (DOCX 15 kb)

## References

[1] Pieters R, de Groot-Kruseman H, Van der Velden V, Fiocco M, van den Berg H, de Bont E, Egeler RM, Hoogerbrugge P, Kaspers G, Van der Schoot E, De Haas V, Van Dongen J (2016). Successful therapy reduction and intensification for childhood acute lymphoblastic leukemia based on minimal residual disease monitoring: study ALL10 from the Dutch Childhood Oncology Group. J Clin Oncol.

[2] Warris LT, van den Heuvel-Eibrink MM, Aarsen FK, Pluijm SM, Bierings MB, van den Bos C, Zwaan CM, Thygesen HH, Tissing WJ, Veening MA, Pieters R, van den Akker EL (2016). Hydrocortisone as an intervention for dexamethasone-induced adverse effects in pediatric patients with acute lymphoblastic leukemia: results of a double-blind, randomized controlled trial. J Clin Oncol.

[3] Stuart FA, Segal TY, Keady S (2005). Adverse psychological effects of corticosteroids in children and adolescents. Arch Dis Child.

[4] Warris LT, van den Heuvel-Eibrink MM, den Hoed MA, Aarsen FK, Pieters R, van den Akker EL (2014). Does dexamethasone induce more neuropsychological side effects than prednisone in pediatric acute lymphoblastic leukemia? A systematic review. Pediatr Blood Cancer.

[5] McGrath P, Pitcher L (2002). ‘Enough is enough’: qualitative findings on the impact of dexamethasone during reinduction/consolidation for paediatric acute lymphoblastic leukaemia. Support Care Cancer.

[6] McCarthy MC, Bastiani J, Williams LK (2016). Are parenting behaviors associated with child sleep problems during treatment for acute lymphoblastic leukemia?. Cancer Med.

[7] Williams LK, Lamb KE, McCarthy MC (2014). Behavioral side effects of pediatric acute lymphoblastic leukemia treatment: the role of parenting strategies. Pediatr Blood Cancer.

[8] Long KA, Keeley L, Reiter-Purtill J, Vannatta K, Gerhardt CA, Noll RB (2014). Child-rearing in the context of childhood cancer: perspectives of parents and professionals. Pediatr Blood Cancer.

[9] Warris LT, van den Akker EL, Aarsen FK, Bierings MB, van den Bos C, Tissing WJ, Sassen SD, Veening MA, Zwaan CM, Pieters R, van den Heuvel-Eibrink MM (2016). Predicting the neurobehavioral side effects of dexamethasone in pediatric acute lymphoblastic leukemia. Psychoneuroendocrinology.

[10] Meijer OC, de Kloet ER (2017). A refill for the brain mineralocorticoid receptor: the benefit of cortisol add-on to dexamethasone therapy. Endocrinology.

[11] Steiger A (2002). Sleep and the hypothalamo-pituitary-adrenocortical system. Sleep Med Rev.

[12] Luik AI, Direk N, Zuurbier LA, Hofman A, Van Someren EJ, Tiemeier H (2015). Sleep and 24-h activity rhythms in relation to cortisol change after a very low-dose of dexamethasone. Psychoneuroendocrinology.

[13] Pesonen AK, Martikainen S, Kajantie E, Heinonen K, Wehkalampi K, Lahti J, Strandberg T, Raikkonen K (2014). The associations between adolescent sleep, diurnal cortisol patterns and cortisol reactivity to dexamethasone suppression test. Psychoneuroendocrinology.

[14] Raikkonen K, Matthews KA, Pesonen AK, Pyhala R, Paavonen EJ, Feldt K, Jones A, Phillips DI, Seckl JR, Heinonen K, Lahti J, Komsi N, Jarvenpaa AL, Eriksson JG, Strandberg TE, Kajantie E (2010). Poor sleep and altered hypothalamic-pituitary-adrenocortical and sympatho-adrenal-medullary system activity in children. J Clin Endocrinol Metab.

[15] Kellner M, Wiedemann K (2008). Mineralocorticoid receptors in brain, in health and disease: possibilities for new pharmacotherapy. Eur J Pharmacol.

[16] Zupanec S, Jones H, Stremler R (2010). Sleep habits and fatigue of children receiving maintenance chemotherapy for ALL and their parents. J Pediatr Oncol Nurs.

[17] Daniel LC, Li Y, Kloss JD, Reilly AF, Barakat LP (2016). The impact of dexamethasone and prednisone on sleep in children with acute lymphoblastic leukemia. Support Care Cancer.

[18] van Litsenburg RR, Huisman J, Hoogerbrugge PM, Egeler RM, Kaspers GJ, Gemke RJ (2011). Impaired sleep affects quality of life in children during maintenance treatment for acute lymphoblastic leukemia: an exploratory study. Health Qual Life Outcomes.

[19] Rogers VE, Zhu S, Ancoli-Israel S, Hinds PS (2014). Impairment in circadian activity rhythms occurs during dexamethasone therapy in children with leukemia. Pediatr Blood Cancer.

[20] Barsevick AM, Irwin MR, Hinds P, Miller A, Berger A, Jacobsen P, Ancoli-Israel S, Reeve BB, Mustian K, O'Mara A, Lai JS, Fisch M, Cella D, National Cancer Institute Clinical Trials Planning M (2013). Recommendations for high-priority research on cancer-related fatigue in children and adults. J Natl Cancer Inst.

[21] Nunes MDR, Jacob E, Adlard K, Secola R, Nascimento LC (2015). Fatigue and sleep experiences at home in children and adolescents with Cancer. Oncol Nurs Forum.

[22] Hinds PS, Hockenberry MJ, Gattuso JS, Srivastava DK, Tong X, Jones H, West N, McCarthy KS, Sadeh A, Ash M, Fernandez C, Pui CH (2007). Dexamethasone alters sleep and fatigue in pediatric patients with acute lymphoblastic leukemia. Cancer.

[23] Nap-van der Vlist MM, Dalmeijer GW, Grootenhuis MA, van der Ent CK, van den Heuvel-Eibrink MM, Wulffraat NM, Swart JF, van Litsenburg RRL, van de Putte EM, Nijhof SL (2019). Fatigue in childhood chronic disease. Arch Dis Child.

[24] Knight SJ, Politis J, Garnham C, Scheinberg A, Tollit MA (2018). School functioning in adolescents with chronic fatigue syndrome. Front Pediatr.

[25] Salter A, Fox RJ, Tyry T, Cutter G, Marrie RA (2019). The association of fatigue and social participation in multiple sclerosis as assessed using two different instruments. Mult Scler Relat Disord.

[26] Rogers VE, Zhu S, Mandrell BN, Ancoli-Israel S, Liu L, Hinds PS (2019). Relationship between circadian activity rhythms and fatigue in hospitalized children with CNS cancers receiving high-dose chemotherapy. Support Care Cancer.

[27] Standaard Onderwijsindeling 2016 [Standard Educational Classification] (2016) Den Haag/Heerlen: Centraal Bureau voor de Statistiek [Statistics Netherlands]

[28] Ancoli-Israel S, Cole R, Alessi C, Chambers M, Moorcroft W, Pollak CP (2003). The role of actigraphy in the study of sleep and circadian rhythms. Sleep.

[29] Sadeh AA, C. (2002). The role of actigraphy in sleep medicine. Sleep Med Rev.

[30] Sadeh A, Hauri PJ, Kripke DF, Lavie P (1995). The role of actigraphy in the evaluation of sleep disorders. Sleep.

[31] Luik AI, Zuurbier LA, Hofman A, Van Someren EJ, Tiemeier H (2013). Stability and fragmentation of the activity rhythm across the sleep-wake cycle: the importance of age, lifestyle, and mental health. Chronobiol Int.

[32] Mitchell JA, Quante M, Godbole S, James P, Hipp JA, Marinac CR, Mariani S, Cespedes Feliciano EM, Glanz K, Laden F, Wang R, Weng J, Redline S, Kerr J (2017). Variation in actigraphy-estimated rest-activity patterns by demographic factors. Chronobiol Int.

[33] van Someren EJW, Swaab DF, Colenda CC, Cohen W, McCall WV, Rosenquist PB (1999). Bright light therapy: improved sensitivity to its effects on rest-activity rhythms in Alzheimer patients by application of nonparametric methods. Chronobiol Int.

[34] Rensen N, Steur LMH, Wijnen N, Van Someren EJW, Kaspers GJL, Van Litsenburg RRL (2020) Actigraphic estimates of sleep and the sleep-wake rhythm, and 6-sulfatoxymelatonin levels in healthy Dutch children. Chronobiol Int 10.1080/07420528.2020.1727916 32126835

[35] Gordijn M, Cremers EM, Kaspers GJ, Gemke RJ (2011). Fatigue in children: reliability and validity of the Dutch PedsQL Multidimensional Fatigue Scale. Qual Life Res.

[36] Varni JW, Burwinkle TM, Katz ER, Meeske K, Dickinson P (2002). The PedsQL in pediatric cancer: reliability and validity of the Pediatric Quality of Life Inventory Generic Core Scales, Multidimensional Fatigue Scale, and Cancer Module. Cancer.

[37] Hofstra WA, de Weerd AW (2008). How to assess circadian rhythm in humans: a review of literature. Epilepsy Behav.

[38] Duffy JF, Wright KP (2005). Entrainment of the human circadian system by light. J Biol Rhythm.

[39] Baron KG, Reid KJ (2014). Circadian misalignment and health. Int Rev Psychiatry.

[40] Scheer FA, Pirovano C, Van Someren EJ, Buijs RM (2005). Environmental light and suprachiasmatic nucleus interact in the regulation of body temperature. Neuroscience.

[41] Rosen G, Harris AK, Liu M, Dreyfus J, Krueger J, Messinger YH (2015). The effects of dexamethasone on sleep in young children with acute lymphoblastic leukemia. Sleep Med.

[42] Vallance K, Liu W, Mandrell BN, Panetta JC, Gattuso JS, Hockenberry M, Zupanec S, Yang L, Yang J, Hinds PS (2010). Mechanisms of dexamethasone-induced disturbed sleep and fatigue in paediatric patients receiving treatment for ALL. Eur J Cancer.

[43] Daniel LC, Brumley LD, Schwartz LA (2013). Fatigue in adolescents with cancer compared to healthy adolescents. Pediatr Blood Cancer.

[44] Moyer-Mileur LJ, Ransdell L, Bruggers CS (2009). Fitness of children with standard-risk acute lymphoblastic leukemia during maintenance therapy: response to a home-based exercise and nutrition program. J Pediatr Hematol Oncol.

[45] McDowall PS, Elder DE, Campbell AJ (2017). Relationship between parent knowledge of child sleep, and child sleep practices and problems: a pilot study in a children's hospital cohort. J Paediatr Child Health.

[46] Wilson KE, Miller AL, Bonuck K, Lumeng JC, Chervin RD (2014). Evaluation of a sleep education program for low-income preschool children and their families. Sleep.

[47] Demoule A, Carreira S, Lavault S, Pallanca O, Morawiec E, Mayaux J, Arnulf I, Similowski T (2017). Impact of earplugs and eye mask on sleep in critically ill patients: a prospective randomized study. Crit Care.

[48] Gordijn MS, van Litsenburg RR, Gemke RJ, Huisman J, Bierings MB, Hoogerbrugge PM, Kaspers GJ (2013). Sleep, fatigue, depression, and quality of life in survivors of childhood acute lymphoblastic leukemia. Pediatr Blood Cancer.

[49] Acebo C, Sadeh A, Seifer R, Tzischinsky O, Hafer A, Carskadon MA (2005). Sleep/wake patterns derived from activity monitoring and maternal report for healthy 1- to 5-year-old children. Sleep.

