# Supplementary material for: The impact of maintenance therapy on sleep-wake rhythms and cancer-related fatigue in pediatric acute lymphoblastic leukemia
Source: Support Care Cancer. 2020 Apr 13;28(12):5983–93. doi: 10.1007/s00520-020-05444-7 (PMC7686190; doi:10.1007/s00520-020-05444-7)
Supplement: Supplementary file 1 — (DOCX 15 kb) [file 520_2020_5444_MOESM1_ESM.docx]

| Supplemental Table S1. Sleep-wake rhythm outcomes and cancer-related fatigue in MR patients participating in the assessment without dexamethasone versus SR patients (linear regression) | | | |
| --- | --- | --- | --- |
| *Sleep-wake rhythm outcomes* | *B*^a,b^ | *95%-CI* | *P-value* |
| Interdaily stability | 0.02 | -0.04; 0.08 | 0.566 |
| Intradaily variability | 0.07 | -0.10; 0.12 | 0.898 |
| L5 counts | 6.45 | -8.68; 21.58 | 0.398 |
| M10 counts | -103.39 | -448.54; 241.76 | 0.552 |
| Relative amplitude | 0.01 | -0.03; 0.05 | 0.691 |
| *Cancer-related fatigue* | *B*^a,c^ | *95%-CI* | *P-value* |
| General fatigue | -4.46 | -14.16; 5.24 | 0.364 |
| Sleep-rest fatigue | -1.27 | -10.44; 7.90 | 0.784 |
| Cognitive fatigue | 5.03 | -3.65; 13.70 | 0.253 |
| ^a^SR patients are the reference category in the regression models; ^b^Models are adjusted for child age and sex, child’s sleep medication use, time since diagnosis, and highest attained parental educational level; ^c^Models are adjusted for child age and sex, child’s sleep medication use, time since diagnosis, and parental sex and highest attained parental educational level; CI: confidence interval. ALL: acute lymphoblastic leukemia, MR: medium risk, SR: standard risk. | | | |

| Supplemental Table S2. Sleep-wake rhythm variables and cancer-related fatigue in ALL patients participating in the assessment without dexamethasone compared to healthy children (linear regression) | | | |
| --- | --- | --- | --- |
| *Sleep-wake rhythm outcomes* | *B*^a,b^ | *95%-CI* | *P-value* |
| Interdaily stability | -0.04 | -0.07; -0.004 | 0.030 |
| Intradaily variability | 0.05 | -0.01; 0.11 | 0.094 |
| L5 counts | 0.33 | -7.97; 8.62 | 0.938 |
| M10 counts | -412.23 | -600.70; -223.77 | <0.001 |
| Relative amplitude | -0.02 | -0.04; -0.01 | 0.012 |
| *Cancer-related fatigue* | *B*^a,c^ | *95%-CI* | *P-value* |
| General fatigue | -17.52 | -21.05; -13.99 | <0.001 |
| Sleep-rest fatigue | -12.11 | -15.52; -8.71 | <0.001 |
| Cognitive fatigue | 0.54 | -3.52; 4.61 | 0.793 |
| ^a^The reference population is the reference category in regression model; ^b^Models are adjusted for child age, sex, and sleep medication use and highest attained parental educational level; *^c^*Models are adjusted for child age, sex and sleep medication use and parental sex and highest attained parental educational level; ALL: acute lymphoblastic leukemia, CI: confidence interval | | | |

| Supplemental Table S3. Sleep-wake rhythm outcomes and cancer-related fatigue in ALL patients participating in the assessment without dexamethasone versus MR patients participating in the assessment with dexamethasone (mixed models) | | | |
| --- | --- | --- | --- |
| *Sleep-wake rhythm outcomes* | *B*^a,b^ | *95%-CI* | *P-value* |
| Interdaily stability | 0.01 | -0.03; 0.04 | 0.790 |
| Intradaily variability | 0.02 | -0.04; 0.08 | 0.526 |
| L5 counts | -5.56 | -13.23; 2.10 | 0.151 |
| M10 counts | 254.94 | 111.70; 398.19 | 0.001 |
| Relative amplitude | 0.01 | -0.002; 0.03 | 0.081 |
| *Cancer-related fatigue* | *B*^a,c^ | *95%-CI* | *P-value* |
| General fatigue | 10.50 | 4.32; 16.68 | 0.001 |
| Sleep-rest fatigue | 10.63 | 5.46; 15.80 | <0.001 |
| Cognitive fatigue | 9.63 | 4.26; 15.00 | 0.001 |
| ^a^Patient with dexamethasone are the reference category in mixed model analyses; ^b^Models are adjusted for child age and sex, child’s sleep medication use, time since diagnosis, and highest attained parental educational level; ^c^Models are adjusted for child age and sex, child’s sleep medication use, time since diagnosis, and parental sex and highest attained parental educational level; CI: confidence interval. ALL: acute lymphoblastic leukemia, MR: medium risk | | | |
